# Flash 3D Imaging of Far-Field Dynamic Objects: An EMCCD-Based Polarization Modulation System

**DOI:** 10.3390/s25133852

**Published:** 2025-06-20

**Authors:** Shengjie Wang, Xiaojia Yang, Donglin Su, Weiqi Cao, Xianhao Zhang

**Affiliations:** 1 The Key Laboratory of Flight Techniques and Flight Safety, Civil Aviation Flight University of China, Guanghan 618307, China; 2 The Second Research Institute of CAAC, Chengdu 610041, China; 3 Electronic Information Engineering, Beihang University, Beijing 100191, China

**Keywords:** Electron Multiplying CCDs, high-resolution, range-gated imaging

## Abstract

**Highlights:**

**What are the main findings?**

- Polarization-Modulated 3D Imaging: The key novelty is the synergy between polarization modulation and a dual-EMCCD framework. In the dual-cameras structure, the ratio of intensities in one camera versus the other camera provides range information, while the summation of intensities in both cameras provides transverse information. Ultimately, 3D image about the scene can be produced after 3D reconstruction. The advantage of this technique is that we can use a pair of EMCCD cameras for super-resolution 3D imaging, with which a frame of 3D image can be reconstructed from a frame of polarization-modulated images.
- Dual-EMCCD Framework for Dynamic Scene Reconstruction: A dual-EMCCD setup simultaneously reconstructs high-resolution depth images and grayscale intensity images from a single raw data frame. Combined with adaptive gate-opening range technology, the system achieves 10 cm range resolution for specific objects, overcoming motion-induced artifacts in dynamic environments (e.g., moving objects or platforms).

**What is the implication of the main finding?**

- Advancing High-Speed 3D Imaging: The EMCCD’s low bandwidth and rapid shuttering capabilities address the limitations of traditional avalanche photodiode (APD) arrays, enabling ultrafast, noise-resilient 3D imaging for applications requiring real-time depth sensing in dynamic scenarios (e.g., autonomous vehicles, robotics).
- Enhanced Versatility for Complex Environments: The dual-image reconstruction (depth + grayscale) and adaptive range gating provide motion-agnostic imaging performance, making the system robust in scenarios with rapid object/platform movement or cluttered scenes. This innovation could revolutionize applications like remote sensing of fast-moving targets where both spatial and temporal resolution are critical.

**Abstract:**

High-resolution 3D visualization of dynamic environments is critical for applications such as remote sensing. Traditional 3D imaging systems, such as lidar, rely on avalanche photodiode (APD) arrays to determine the flight time of light for each scene pixel. In this context, we introduce and demonstrate a high-resolution 3D imaging approach leveraging an Electron Multiplying Charge Coupled Device (EMCCD). This sensor’s low bandwidth properties allow for the use of electro-optic modulators to achieve both temporal resolution and rapid shuttering at sub-nanosecond speeds. This enables range-gated 3D imaging, which significantly enhances the signal-to-noise ratio (SNR) within our proposed framework. By employing a dual EMCCD setup, it is possible to reconstruct both a depth image and a grayscale image from a single raw data frame, thereby improving dynamic imaging capabilities, irrespective of object or platform movement. Additionally, the adaptive gate-opening range technology can further refine the range resolution of specific scene objects to as low as 10 cm.

## 1. Introduction

Light Detection and Ranging (LiDAR) achieves three-dimensional modeling of a scene by measuring the three-dimensional information of the target. This technology estimates the distance by calculating the time it takes for light to travel to the scene and back, a measurement known as time-of-flight (TOF) [1,2]. Presently, there is a spectrum of TOF measurement systems in use, including direct and coherent detection methods; typically, they vary in their methods of regulating light emission and in conducting measurements that capture changes over time [3]. The primary challenge at hand is to streamline the data collection process, minimize the time required for data acquisition, and ensure comprehensive imaging capabilities, sensitivity in low-light conditions, and preservation of temporal resolution [4].

The Geiger-mode Avalanche Photodiode (GM-APD) array-based LiDAR system was initially developed by MIT/Lincoln Laboratory [5,6]. A limitation of GM-APD is the existence of a non-responsive period, or dead time, post each detection event, during which it is unable to sense any photons. Furthermore, GM-APDs are incapable of measuring the intensity of the signal per pixel, thus failing to produce a grayscale image for each pulse [3]. Building on the foundation of Single-Photon Avalanche Diode (SPAD) detectors, which function in time-correlated single-photon counting (TCSPC) mode, first-photon imaging [7,8] and photon-efficient imaging [9] were developed. The superior temporal resolution of SPAD detectors is beneficial for single-photon time-of-flight measurements, yet their spatial resolution is quite limited, with a resolution of only 32 × 32 pixels. Due to constraints of the readout integrated circuit (ROIC), the maximum array size for SPAD detectors is currently 256 × 256 pixels [10,11]. As a result, achieving high-resolution 3D imaging with SPAD detectors is challenging. To enhance the spatial resolution and reduce the sampling rate for 3D imaging, a digital camera optically linked to a second-generation image Intensified Charge-Coupled Device (ICCD) is used for range-gated imaging, as shown in [12,13,14], achieving a spatial resolution exceeding 512 × 512 pixels. However, the ICCD imaging process, which involves multiple photon–electron conversions, limits its spatial resolution compared to a non-intensified camera with the same CCD chip and results in a lower quantum efficiency (QE) than a bare CCD [15,16]. Additionally, the signal-to-noise ratio of ICCD is heavily influenced by the quantum efficiency and noise characteristics of the micro-channel plate (MCP).

In the present study, we introduce and conduct experimental validation of a 3D imaging framework with high resolution, which is capable of substantially enhancing the transverse resolution. The technique we employ is reliant on the EMCCD camera, which has a transverse resolution of 1024 × 1024 pixels. Although the SNR performance of the EMCCD camera is significantly affected by thermal dark current noise, and this noise is amplified along with the signal via the gain register, a cooled EMCCD can achieve the best image quality and outperform ICCD as documented in [17,18,19]. Additionally, our 3D imaging framework attains high resolution through the process of single photon–electron conversion without the need to integrate other devices. As a result, it is able to offer enhanced transverse resolution and improved quantum efficiency. In the structure of the three-dimensional imaging system, a high-speed electro-optic modulator (EOM) provides high-precision control over temporal (range) resolution.

## 2. Materials and Methods

### 2.1. 3D Imaging Setup

**Figure 1:**
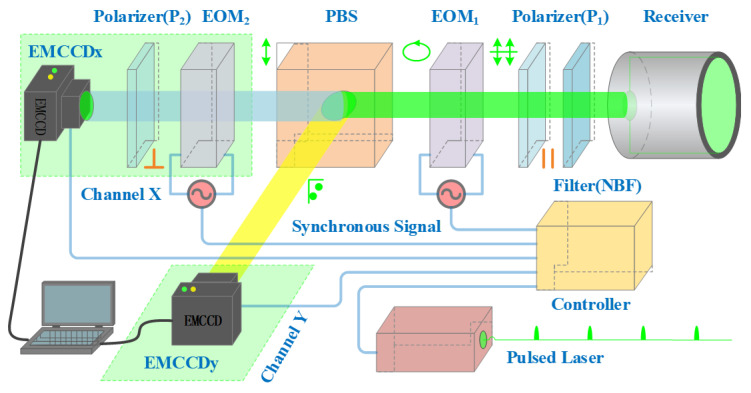
High-resolution 3D imaging system. In this system architecture, the return light first passes through the first electro-optic modulator (EOM1), and the polarization state of the return light is altered for time-resolved imaging. Meanwhile, the second modulator (EOM2) serves as a high-speed shutter to achieve range-gated imaging. The two electron-multiplying charge-coupled device (EMCCD) cameras are combined to receive the polarization-modulated images of channel X and channel Y, respectively, and complete the 3D reconstruction of the target.

Our experimental configuration for 3D Imaging is outlined in Figure 1. The system employs a pulsed laser as the light source, which operates at a frequency of 10 Hz with a high pulse energy of 200 mJ and a pulse width of 8 ns. The light beam, which is linearly polarized, is projected towards the target scene, where it interacts with objects by reflection or scattering. Upon the return of the light to the receiver, a narrowband filter (NBF) is used to filter out background radiation from sources like sunlight. A linear polarizer (P1), aligned with the polarization of the emitted light, is crucial for filtering out light that is not parallel to the original polarization, thus retaining only the desired linearly polarized component. Through the utilization of a fluctuating voltage on the electro-optic modulator (EOM 1) [20], a phase shift that hinges on the voltage value emerges, which manages to transform the linearly polarized illumination into elliptically polarized illumination. Subsequently, this light is segregated into its perpendicular constituents, namely the p-polarized and s-polarized ones, with the assistance of a polarization beam splitter (PBS). For the detection of both polarizations, it is feasible to use a pair of EMCCD cameras. The p-polarized light (Blue light beam) is received by EMCCDx via Channel X. Meanwhile, the s-polarized light (Yellow light beam) is received by EMCCDy via Channel Y.

In a dual-camera system, the disparity in intensity levels captured by each camera provides crucial range data. Concurrently, the sum of intensities within both cameras offers transverse information. Subsequently, following the process of 3D reconstruction, a 3D image of the scene can be successfully produced. This technology presents distinctive luminance. The advantage of this technique is that we can use a pair of EMCCD cameras for high-resolution 3D imaging. With just one single frame of polarization-modulated imagery, it can extract and reconstruct the three-dimensional details of the target, facilitating swift and highly accurate three-dimensional imaging of moving objects. Such an advantage endows the 3D imaging framework with the capacity to operate as a lidar system, rendering it particularly well-suited for dynamic 3D imaging applications.

**Figure 2:**
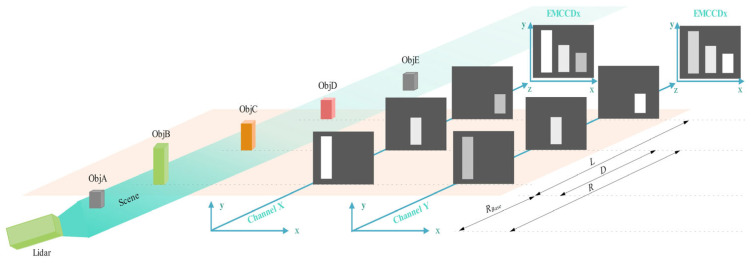
Synchronous image acquisition for range-gated imaging. As the electro-optic modulator is able to act as a fast shutter, this system can selectively receive the returned light to achieve range-gated imaging, which can effectively improve the signal-to-noise ratio performance. Here, there are five objects (including ObjA, ObjB, ObjC, ObjD, and ObjE) located at different positions, and ObjB, ObjC, and ObjD are located inside the gate opening range (L) while ObjA and ObjD are located outside the gate opening range. Therefore, only the returned light reflected by ObjB, ObjC, and ObjD can arrive at the EMCCD cameras. In channel X, the near-range ObjB modulated to be brighter, while the far-range ObjD is converted into a dark one. The converse situation occurs in channel Y, such that the near-range object is rendered as a dark representation and the far-range object as a bright one.

Range-gated imaging is widely recognized for its ability to efficiently suppress backscatter from diverse mediums, encompassing air molecules, fog, camouflage materials, and aquatic environments [21,22,23]. Within our framework, deploying range-gated imaging in channel X requires the integration of an extra electro-optic modulator (EOM2) and a linear polarizer (P2, positioned at a right angle to the initial linearly polarized light). They are placed between the PBS and the EMCCDx camera. After the return light passes through the EOM1 and is split by the PBS, they control when the p-polarized light passes through channel X and for how long. In contrast to channel X, channel Y eliminates the requirement for additional components. The existing setup of EOM1 and PBS is fully capable of managing and specifying the exact timing and duration for s-polarized light to pass through channel Y. When appropriate voltages are precisely applied to the two electro-optic modulators, EOM1 and EOM2 respectively, the p-polarized light and the s-polarized light can not only be isolated from each other without interference, but also synchronously pass through both channels to achieve a special optical path layout. In conclusion, the deployment of range-gated imaging is successfully realized, offering a measurement capability that spans from *R_Base_* to *R_Base_* + *L* (see Figure 2), thereby providing a comprehensive depth resolution.

### 2.2. Image Acquisition and Processing

As depicted in Figure 1, for the purpose of guaranteeing the accurate modulation and accumulation of the returned light, synchronous signals are essential to appropriately manage the pulsed laser, electro-optic modulators, and the EMCCD cameras. The lidar system emits a pulse of light toward the target scene and then receives the return light. The return light is captured by two EMCCD cameras within 0.1 s. This frame of raw data is directly transmitted to a computer for processing, and the three-dimensional image of the target is calculated and reconstructed. Even though the frame rate of the cameras is 10 Hz, the exposure time is merely 0.32 microseconds (μs). Therefore, even if an object is moving at a speed of 100 m/s within the field of view of the system, its lateral displacement is only 30 μm. Thus, a high-speed moving target can be regarded as stationary for this system.

Dual EMCCDs operate in an external exposure triggering mode, where the exposure duration is precisely regulated by an external trigger signal. During the period when the trigger signal is high, the EMCCD accumulates charge within the image region. Upon the trigger signal transitioning to low, the accumulated charge is swiftly transferred to the storage region and subsequently read out with significant electron-multiplying gain. Nevertheless, a disadvantage of the frame-transfer architecture in EMCCD camera is the charge smearing, which is caused by the phenomenon that the returned light falls on the image area whilst accumulated charge is being transferred to the storage area. To avoid this, utilizing a mechanical shutter to cover the image area is necessary during readout procedure. However, mechanical shutters have lifetime issues and are too slow to afford a fast exposure time with sub-microsecond level. Fortunately, the electro-optic modulator is able to act as a fast shutter for the EMCCD camera to provide an exposure time with sub-microsecond level, which can prevent charge smearing happening effectively.

As previously mentioned, the range information is obtained from the ratio of intensities between one camera and the other camera. Regrettably, the gray level that represents the intensity of light within the camera is prone to being affected by diverse types of noise, including readout noise, poisoning shot noise, dark current noise, and so on. Such interference will, to some extent, undermine the range accuracy. In the case of the EMCCD camera, the SNR (Signal-to-Noise Ratio) performance can be expressed as

(1) $$SNR=\frac{Q_{E}\cdot S}{\sqrt{F^{2}\left(Q_{E}\cdot S+N_{Dark}^{2}\right)+\frac{N_{Readout}^{2}}{M^{2}}}}$$

where S represents the signal photons of returned-light, $Q_{E}$ is the quantum efficiency, F is the noise factor, M is the electron multiplying (EM) gain, $N_{Readout}$ is the readout noise, and $N_{Dark}$ is the dark current noise caused by thermally generated electrons in the silicon substrate of the CCD. To enhance SNR performance and boost the sensitivity of the EMCCD camera for low-light-level applications, the subsequent steps should be carried out: initially, cooling the EMCCD chip to a temperature as low as −60 °C or even lower to reduce the thermal noise to a negligible level, which will significantly diminish the dark current noise. Subsequently, setting the EM gain to 300 or a higher value enables the signal to be amplified before being read out. This relatively lessens the impact of $N_{Readout}$ and improves the signal-to-noise ratio without impeding the readout rate. Finally, an image denoising algorithm is utilized to eliminate the signal-dependent noise ($\sqrt{Q_{E}\cdot S}$) and other uncertain noise [23,24,25].

Furthermore, to accurately reconstruct both the depth and intensity images from the polarization-modulated images captured by the dual EMCCD cameras, precise sub-pixel image registration is essential. This process aligns the pixels of one image with the corresponding pixels of the other [26,27,28,29]. After sub-pixel registration, although the two EMCCD cameras are slightly misaligned, the alignment accuracy will still be better than one pixel.

### 2.3. 3D Structure Reconstruction

Within the realm of three-dimensional visualization, a device utilizing the electro-optical properties of crystalline materials is implemented to enable temporally resolved image acquisition. This apparatus, known as an electro-optical modulator, harnesses the inherent electro-optical phenomenon exhibited by certain crystals to achieve precise temporal control in imaging applications. Upon applying a ramp voltage to the modulator along the light’s propagation path, a phase retardation effect occurs between the ordinary and extraordinary waves. The magnitude of this phase delay exhibits a linear relationship with the magnitude of the applied electrical potential. This direct correlation between the retardation and the input voltage demonstrates a proportional dependence, where increasing the electrical potential results in a corresponding increase in the optical retardation. Given that objects at varying distances produce distinct round-trip times, the phase retardation, denoted as θ, can be expressed as a function of range.

(2) $$\theta =\pi \cdot \frac{D}{L},\left(0\leq D\leq L\right)$$

In this context, *L* denotes the extent of the gate’s opening, which is influenced by the time duration for which the range-gate remains open, while *D* signifies the distance from the object to the initial point of the gate’s range (as shown in Figure 2). Such phase retardation will give rise to distinct intensity distributions in channel *X* and *Y*, respectively [30].

(3) $$\left\{\begin{array}{c} I_{X}=I_{REC}\cos^{2}\left(\frac{\pi }{2}\cdot \frac{D}{L}\right)\\ I_{Y}=I_{REC}\sin^{2}\left(\frac{\pi }{2}\cdot \frac{D}{L}\right) \end{array}\right.,\left(0\leq D\leq L\right)$$

The strength of the reflected light is denoted as $I_{REC}$, while $I_{X}$ and $I_{Y}$ represent the strengths of the p-polarized and s-polarized light components, respectively. It is evident that the polarization-modulated images, derived from Equation (3), encompass depth data, which allows for the execution of 3D reconstruction using the aforementioned formula. As a result, the range *R* from the lidar system to the target object can be calculated using the following formula:

(4) $$R=R_{Base}+\frac{2L}{\pi }\arctan\left(\sqrt{\frac{I_{Y}}{I_{X}}}\right)$$

Additionally, Equation (3) indicates that the summation of components $I_{X}$ and $I_{Y}$ will result in a polarization-demodulated image, which is equivalent to a conventional grayscale image, as described below.

(5) $$I_{X}+I_{Y}=I_{REC}$$

Range resolution is proportional to phase resolution and can be derived from the following expression:

(6) $$\Delta R=\frac{2L}{\pi }\Delta \theta =\frac{2L}{\pi }\arctan\left(\sqrt{\frac{1}{2^{n}}}\right)$$

where Δθ is the phase resolution, and n is the resolution of analog-to-digital converter (ADC) in bits. For a 16-bit ADC (n=16), an EMCCD camera offers 65,536 discrete grayscale values 2^16^. Furthermore, with a gate opening time of 0.32 microseconds, the specified gate opening range is 48 m. Consequently, our 3D imaging system attains a range resolution of roughly 10 cm.

We can also know from Equation (6) that the gate opening range (L) determines directly the range resolution. As a result, higher range resolution (lower ΔR) can be achieved through compressing the gate opening range when given the phase resolution Δθ. Thus, an adaptive range-gated imaging can be designed to improve the range resolution in the 3D imaging framework.

When the duration of the gate opening is dynamically modified, the corresponding gate opening range decreases, leading to improved range resolution. Generally, adaptive range-gated imaging operates in two phases: coarse range imaging and fine range imaging. During the first phase, a broad gate opening range is utilized to identify objects within the field of view and approximate their distances. In the second phase, the gate’s starting position $R_{Base}$ and the gate opening range L are fine-tuned to focus on the target object. As a result, a depth image with higher range resolution can be reconstructed using polarization-modulated images acquired within a more confined gate opening range.

**Figure 3:**
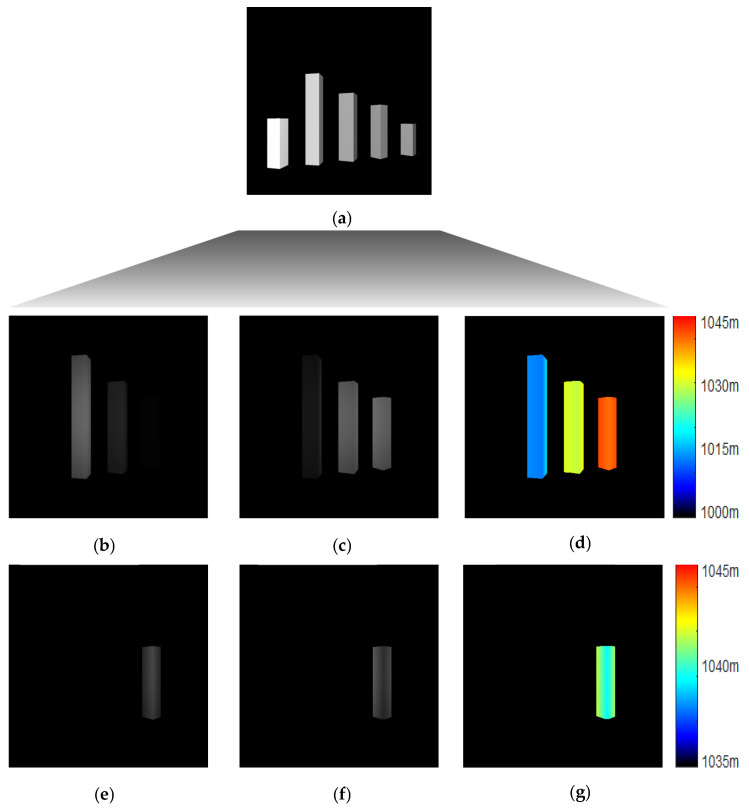
3D structure reconstruction from a frame of raw data. (**a**) represents the grayscale image obtained from a standard CCD camera; (**b**,**c**) are the gray images modulated by cos^2^ and sin^2^ function under the gate opening range of 45m, respectively; (**d**) is the depth image reconstructed from (**b**,**c**); Similarly, (**e**,**f**) depict the grayscale images modulated by the cos^2^ and sin^2^ functions, respectively, within a gate opening range of 10 m. (**g**) is the depth image reconstructed from (**e**,**f**). It can be seen from these figures that only the objects enclosed in the gate opening range can be shown in the gray and depth images.

As shown in Figure 3a, all of the objects in the scene (including ObjA, ObjB, ObjC, ObjD, and ObjE; see Figure 2) appear in the conventional image. The system’s distance to the objects varies between 970 m and 1070 m. In the coarse range imaging stage, a gate opening range of 45 m (spanning from 1000 m to 1045 m) is employed to identify and locate objects. As shown in Figure 3b–d, ObjB, ObjC, and ObjD are visible in the images captured with this wide gate opening range. From the data obtained during coarse range imaging, it is evident that ObjB is approximately between 1005 m and 1020 m; ObjC is roughly between 1020 m and 1035 m; and ObjD extends from 1035 m to 1045 m. In Figure 3d, each object is displayed in a nearly identical color, suggesting that the gate opening range applied during the coarse range imaging phase is too wide to deliver adequate resolution for distinguishing individual objects. As a result, fine range imaging with a more confined gate opening range is necessary to acquire a depth image with greater detail.

To investigate ObjD, fine range imaging with a narrow gate opening range of 10 m (from 1035 m to 1045 m) can be conducted. The outcomes are displayed in Figure 3e–g, where only ObjD is visible and represented in the depth image. In comparison to Figure 3d, the range resolution in Figure 3g has significantly improved, allowing for the extraction of more detailed structural information. As the gate opening range being compressed, the dark current noise and the backscatter from the medium accumulated by the EMCCD cameras will be weaken so that the SNR performance can be improved effectively. However, the depth of scene will also be limited at the same time. Consequently, a reasonable strategy, either outstanding range accuracy or large depth of scene, needs to be made according to the detailed applications.

## 3. Results

**Figure 4:**
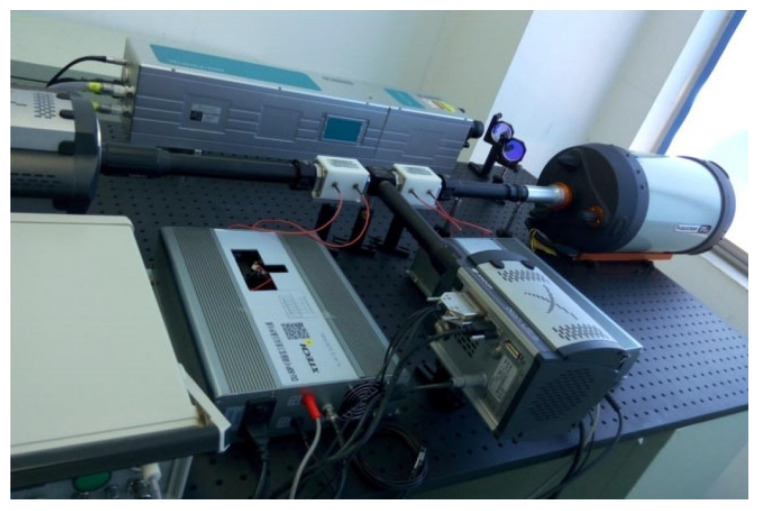
Experimental setup for high-resolution 3D imaging.

The experimental setup for high-resolution 3D imaging is depicted in Figure 4, allowing for the reconstruction of a complete depth image within a single pulsed cycle. During the gate opening interval, two EMCCDs, each with a resolution of 1024 × 1024 pixels, are used to collect the returning light in channels X and Y, respectively. In this configuration, electro-optic modulators perform two essential roles: they act as fast shutters for the EMCCD cameras and as polarization-modulated devices for time-resolved imaging. The key parameters of the 3D imaging system are summarized in Table 1.

The 3D imaging experiments consist two parts: static objects exploration and dynamic objects exploration. In the first experiment, the following steps must be carried out: firstly, place the targets on the roof of the distant building, and adjust the lidar system slightly to align the receiver’s field-of-view with static objects for detection purposes; secondly, steer the laser beam to ensure that field-of-view of the transmitter matches that of the receiver; thirdly, adjust the gate beginning range ($R_{Base}$) to enclose the objects in the gate opening range. Here, the gate beginning range is set to 930 m and therefore the gate opening range (45 m) ranges from 930 m to 975 m; finally, acquire the polarization-modulated images using dual EMCCDs cameras during the gate opening time and then reconstruct the objects’ 3D structure from the polarization-modulated images.

**Figure 5:**
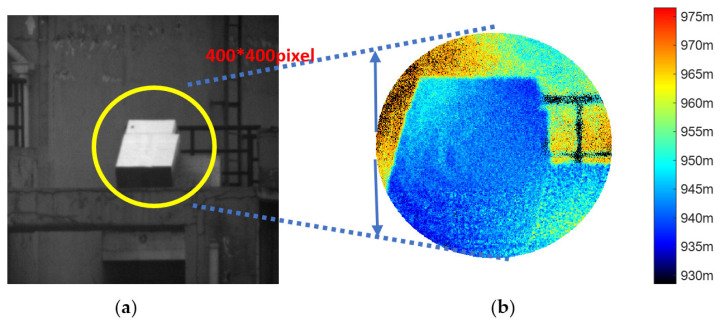
The 3D structure of a box captured by the 3D imaging lidar system is presented. (**a**) The grayscale image obtained using a conventional CCD camera under daylight conditions; (**b**) the depth image, corresponding to the circular region in the grayscale image, is reconstructed using two modulated images. The experiment was conducted at night.

The experimental results are shown in Figure 5. There are two boxes in the scene, one of which is located behind the other. As a result, it can be clearly seen that the front box is leaning against the back box. The grayscale image shown in Figure 5a was captured by a standard CCD camera during daylight hours. Additionally, the depth image depicted in Figure 5b is derived from polarization-modulated images. The colorbar represents the gate opening range, where different colors correspond to the distances from the lidar system to the objects.

The rusted guardrail’s reflected light was extremely weak, causing the EMCCD to fail to capture any return light. As a result, the corresponding pixel intensity registered zero (the black appearance indicates absence of light intensity, not distance information). The estimated depth is obtained from the lidar system while the reference depth is derived from a precise range finder. It’s obvious that the estimated depth meets the reference depth better. Thus, it can be indicated that the experimental results show outstanding range resolution of the 3D imaging framework.

**Figure 6:**
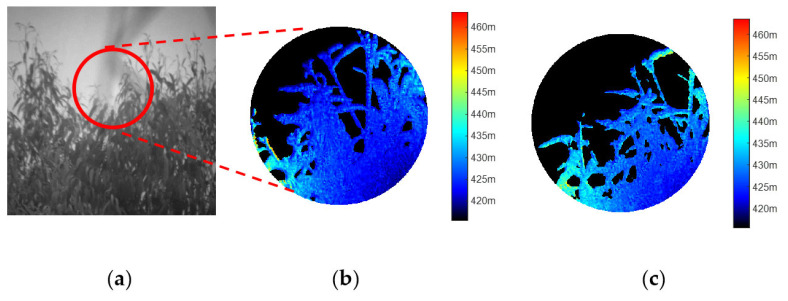
The 3D structure about the tip of a tree located 420 m away from the 3D imaging lidar system. (**a**) is the gray image obtained from conventional CCD camera during the daytime; (**b**,**c**) are two continuous frame of depth images, corresponding roughly to the circular area in the gray image. The experimental work is also implemented at night. It should be noticed that the 3D structures in (**b**,**c**), and the circular area in the gray image are given a little difference. The main reason is that the experiment is implemented under winding condition, which makes the foliage and branches swing all the time.

Similar steps are taken to explore the dynamic objects in the second experiment. As depicted in Figure 6a, multiple trees are visible within the field-of-view. To enclose the trees (about 420 m away from the lidar system) in the gate opening range, the gate beginning range is set to 415.5 m and therefore the gate opening range (48 m) spans from 415.5 m to 463.5 m. The experiment is implemented in winding conditions, so it will result that there is a little difference between one frame of image and another (see Figure 6b,c). With the exposure time being at the sub-second level within a single frame period, the swaying foliage and branches appear as stationary objects. Consequently, a single frame of a 3D image of moving objects can be captured with excellent lateral resolution. Figure 6b,c demonstrates that the foliage and branches are reconstructed clearly, without any ambiguous areas. In fact, high-quality depth images can still be obtained from our 3D imaging framework, even when both the objects and the platform are in motion. Therefore, it can be concluded that the 3D imaging setup is capable of performing dynamic 3D imaging, regardless of whether the objects or the platform are moving.

Due to the high sensitivity characteristics, low-light-level applications are feasible for the EMCCD cameras. In our experimental work, the reconstruction results demonstrate that the EMCCD-based 3D imaging framework can provide the ability for long range detection.

## 4. Discussion

A high-resolution 3D imaging framework for dynamic objects at long range is proposed and demonstrated in this paper. A new imaging sensor EMCCD is introduced in the system, which could provide higher transverse resolution and higher sensitivity for long range applications. By utilizing a dual EMCCD camera setup, 3D reconstruction can be achieved in real time, significantly improving the capability for dynamic imaging, whether dealing with moving objects or platform motion. To achieve temporal (range) resolution for the low-bandwidth camera, an electro-optic modulator is utilized as a time-resolved device by controlling the polarization state of the returning light, the other acts as a fast shutter for the EMCCD camera, which could provide an exposure time with sub-nanosecond level and implement range-gated imaging. Additionally, through adaptive range gate control, the depth image can be “zoomed in” along the range dimension, enabling enhanced focus on specific areas of interest, which promotes the range resolution greatly and provides the ability for a scene’s 3D structure exploration. In addition, the potential application of our 3D imaging framework could be used to identify natural objects or man-made objects by measuring the polarization state of returned light, because such polarization states carry additional information about the objects.

The system achieves rapid imaging and high resolution through synchronous data acquisition of targets within the field of view, making it particularly suitable for long-range applications with stringent real-time requirements, such as autonomous driving and UAV obstacle avoidance. However, its performance is constrained under strong ambient light conditions, while the high cost of EMCCD array detectors and substantial point cloud data processing demands also limit its widespread adoption.

In comparative analysis of avalanche photodiode (APD), intensified CCD (ICCD), and electron-multiplying CCD (EMCCD) sensors for flash 3D imaging, EMCCD demonstrates superior performance in weak-light scenarios due to its unique combination of single-photon sensitivity, ultra-low noise (enabled by deep cooling), and high quantum efficiency (>90% in visible spectrum). Unlike APD, which suffers from limited spatial resolution (10–50 μm pixel pitch) and saturation issues, or ICCD, which introduces microchannel plate (MCP)-induced noise and spatial distortion, EMCCD achieves sub-micron lateral resolution (8–16 μm pixels) with wide dynamic range and linear response, critical for high-precision depth mapping. While APD excels in high-speed applications (ps-level response) and ICCD in gated imaging, EMCCD’s compatibility with computational algorithms and adaptability to low-light conditions position it as the optimal choice for applications demanding both sensitivity and spatial fidelity. Current limitations, including high cost and moderate frame rates, are offset by its unmatched signal-to-noise ratio (SNR), making EMCCD indispensable for advanced 3D imaging system. Future work will focus on cost reduction and real-time processing enhancements to broaden its applicability. A comparison of different photodetector performances is shown in Table 2.

## Figures and Tables

**Table 1:** System Parameters for high-resolution 3D imaging.

System Parameters	Value
Wavelength	532 nm
Pulse Energy	200 mJ
Pulse Duration	8 ns
Frame Rate	10 Hz
Resolution	1024 × 1024 pixels
Wavelength	532 nm
Background light intensity	0.1–1 lux
Target reflectance	87.3% ± 1.5%

**Table 2:** Comparative Performance Analysis of Photodetectors for Flash 3D Imaging.

**Characteristic/Sensor Type**	**Avalanche Photodiode (APD)**	**Intensified CCD (ICCD)**	**Electron-Multiplying CCD (EMCCD)**
Weak-Light Sensitivity	Moderate (saturation issues)	High (but noise-affected)	Optimal (single-photon sensitivity)
Noise Level	Low	High (MCP-induced noise)	Ultra-low (deep cooling technology)
Quantum Efficiency	Not specified	Not specified	>90% (visible spectrum)
Spatial Resolution	Low (10–50 μm pixel pitch)	Moderate (spatial distortion)	High
Dynamic Response	Limited linearity	Restricted dynamic range	Wide dynamic range & linear response
Core Strength	Ultra-high speed (ps-level response)	Gated imaging capability	Sensitivity + spatial fidelity
Primary Limitation	Low resolution, saturation-prone	MCP noise, spatial distortion	High cost, moderate frame rates
Optimal Use Case	High-speed imaging	Time-gated imaging	Low-light 3D imaging (depth mapping)
Signal-to-Noise Ratio (SNR)	Moderate	MCP noise-limited	Best
System Compatibility	Standard circuitry	Requires sync triggering	Strong

## Data Availability

Due to privacy concerns, the data are not available.

## Abbreviations

The following abbreviations are used in this manuscript:

3D	Three-Dimensional
APD	avalanche photodiode
EMCCD	Electron Multiplying Charge Coupled Device
SNR	signal-to-noise ratio
LiDAR	Light Detection and Ranging
TOF	time-of-flight
GM-APD	Geiger-mode Avalanche Photodiode
SPAD	Single-Photon Avalanche Diode
TCSPC	time-correlated single-photon counting
ROIC	readout integrated circuit
ICCD	Intensified Charge-Coupled Device
QE	quantum efficiency
MCP	micro-channel plate
EOM	electro-optic modulator
NBF	narrowband filter
Obj	object
EM	electron multiplying
